# A Curve-Shaped Beam Bistable Piezoelectric Energy Harvester with Variable Potential Well: Modeling and Numerical Simulation

**DOI:** 10.3390/mi12080995

**Published:** 2021-08-21

**Authors:** Xiaoyu Chen, Xuhui Zhang, Luyang Chen, Yan Guo, Fulin Zhu

**Affiliations:** 1 College of Mechanical Engineering, Xi’an University of Science and Technology, Xi’an 710054, China; 19105016004@stu.xust.edu.cn (X.C.); chenluyang@stu.xust.edu.cn (L.C.); g1014901143@163.com (Y.G.); 20205224060@stu.xust.edu.cn (F.Z.); 2 College of Engineering, Zunyi Normal College, Zunyi 563006, China; 3 Shaanxi Key Laboratory of Mine Electromechanical Equipment Intelligent Monitoring, Xi’an 710054, China

**Keywords:** energy harvesting, curve-shaped configuration, variable potential well, dynamic behavior

## Abstract

To improve the energy harvesting performance of an energy harvester, a novel bistable piezoelectric energy harvester with variable potential well (BPEH-V) is proposed by introducing a spring to the external magnet from a curve-shaped beam bistable harvester (CBH-C). First, finite element simulation was performed in COMSOL software to validate that the curved beam configuration was superior to the straight beam in power generation performance, which benefits energy harvesting. Moreover, the nonlinear magnetic model was obtained by using the magnetic dipoles method, and the nonlinear restoring force model of the curve-shaped beam was acquired based on fitting the experimental data. The corresponding coupled governing equations were derived by using generalized Hamilton’s principle, the dynamic responses were obtained by solving the coupling equations with the ode45 method. Finally, the numerical simulations showed that the proposed harvester can make interwell oscillations easier due to the spring being efficiently introduced to pull down the potential barrier compared with the conventional bistable harvester. Spring stiffness has a great impact on characteristics of the system, and a suitable stiffness contributes to realize large-amplitude interwell oscillations over a wide range of excitation, especially in the low excitation condition.

## 1. Introduction

Wireless sensor, wearable devices, and medical implants have shown their significance in modern society [1]. Powering these low-power devices is usually done through conventional batteries, however, these batteries must be regularly recharged or replaced, which can be very costly and cumbersome [2]. Meanwhile, there are environmental issues when disposing of used batteries after operation [3]. Energy harvesting technology holds great potential to achieve the self-powered operation of these devices. Among the various energy harvesting technologies, electromagnetism, electrostatics, and piezoelectricity are the three main methods that generate energy from vibration [4,5]. In particular, vibration-based piezoelectric energy technology can convert kinetic energy from the ambient environment via piezoelectric effect to electric energy, which has received considerable interest for its high energy density, ease of implementation, and miniaturization [6].

At the early stage, research on piezoelectric energy harvesters was mainly based on a linear piezoelectric energy harvester. The linear piezoelectric energy harvester has a high resonance frequency, and when the environmental frequency deviates from its resonance frequency, the power generation performance of the system will drop sharply, resulting in low environmental adaptability [7]. Currently, nonlinear bistable piezoelectric energy harvesters have received great attention. Zhang et al. [8] proposed an arched composite beam magnetically coupled piezoelectric energy harvester. Experiments showed that the effective bandwidth of the energy harvester under magnetic coupling was 3.1 times the bandwidth without magnetic force. Rubes et al. [9] conducted research on magnetically coupled bistable piezoelectric energy harvesters, and their research showed that the introduction of nonlinear stiffness can greatly improve the energy harvesting performance of piezoelectric energy harvesters; Erturk and Inman [10] experimentally proved that the nonlinearity of magnetic coupling can cause vibration between the bistable high-energy traps, thereby improving the collection performance of the energy harvester; and Stanton et al. [11] established a complete dynamic model for the output voltage and dynamic behavior of the magnetic coupling bistable piezoelectric energy harvester and proved the availability of the bistable harvester. Under the condition of simple harmonic excitation, Li et al. [12] developed a magnetic-coupled bi-stable flutter-based energy harvester and proved that the proposed system was an effective design approach for enhancing energy harvesting capability in a low air speed range. Singh et al. [13] investigated a bistable piezoelectric energy harvester with SSHI circuit, and their experiments proved that the output power of the bistable piezoelectric energy harvester with the SSHI circuit reached 478 μw, while the corresponding linear structure was only 129 μw.

The above research shows that the bistable piezoelectric energy harvester is effective for improving the performance of an energy harvester. However, the above harvesters all had a fixed barrier height. In practical applications, the excitation level must provide enough energy to overcome the barrier to achieve a large response, otherwise it will not be able to work well, resulting in poor output performance. In order to reduce the barrier height to improve the performance of the bistable piezoelectric energy harvester, many scholars have carried out studies on piezoelectric energy harvesters with variable potential wells. Zhou et al. [14] placed an external magnet in the middle of the fixed beams at both ends and proposed a bistable system with variable potential wells. Experiments proved that the system not only had a low interwell jump threshold, but also produced higher voltage output. Cao et al. [15] proposed a bistable energy harvesting with time varying potential energy to harvest energy from human motion and various motion speed treadmill tests were performed to demonstrate the advantage of time-varying bistable harvesters over linear and monostable. Nguyen et al. [16] proposed a bistable piezoelectric energy harvester with an auxiliary magnet oscillator and their research showed that this design could improve 114–545% bandwidth compared with traditional bistable piezoelectric energy harvesters. Yang et al. [17,18,19] designed a double-beam piezoelectric energy harvester with variable potential well structure and verified its advantages over traditional bistable piezoelectric energy harvester under random excitation conditions. Lan et al. [20] significantly reduced the barrier height of the traditional bistable piezoelectric energy harvester by adding a small magnet to a traditional bistable energy harvester and compared their design with a three-stable piezoelectric energy harvester, verifying the validity of the proposed device. Shan et al. [21] designed an elastically connected bistable piezoelectric energy harvester based on the straight beam configuration, where the energy harvester had a variable potential barrier during the vibration process. It was experimentally proven that the energy harvesting bandwidth was 60% higher than that of the traditional energy harvester. Li et al. [22] carried out theoretical analysis on the elastically connected straight beam piezoelectric energy harvester, and the results showed that the spring-connected bistable piezoelectric energy harvester had a variable potential function and better energy harvesting performance under low-frequency excitation. Kim et al. [23] designed a multi-degree of freedom (MDOF) vibration energy harvesting system that leverages magnetically coupled bistable and linear harvesters, where the analytical, numerical, and experimental investigations revealed that the novel harvester could facilitate the energetic interwell response for relatively low excitation amplitudes and frequencies by passively and adaptively lowering the potential energy barrier level. Qian et al. [24] developed a broadband piezoelectric energy harvester (PEH) with a mechanically tunable potential function, and the simulations proved that the proposed PEH could harvest vibration energy in a wide frequency range of 0–91 Hz at the excitation level of 0.5 g.

Inspired by the development of variable-potential-energy techniques, this paper proposes a novel bistable energy harvester with a variable potential well. Meanwhile, we used a curve-shaped beam as the energy transducing element to further improve the performance of the piezoelectric energy harvester due to the disadvantages of the straight beam in terms of uneven stress, low conversion efficiency [25,26]. The finite element simulation was performed for the curve-shaped beam and the conventional beam. The results show that the curved beam structure has a special stress distribution and can improve output voltage compared with the straight beam structure. Then, the dynamic model of BPEH-V system is established. Numerical simulation analysis showed that it was easier for the proposed harvester to achieve large-amplitude response in a low-frequency environment compared with the conventional counterpart, and the spring stiffness had an important impact on system performance. The research can provide theoretical guidance for the optimal design and engineering application of the novel piezoelectric energy harvester.

## 2. Finite-Element Simulation

### 2.1. Stress Analysis

**Figure 1:**
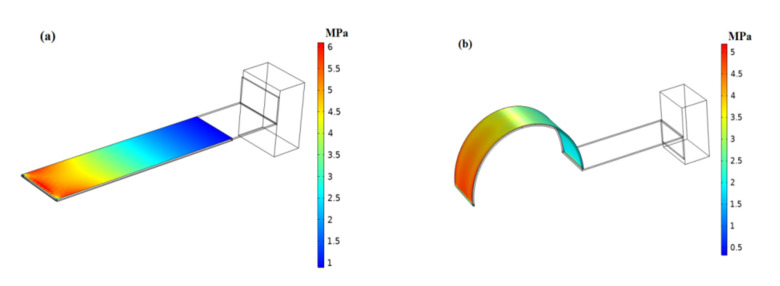
Finite-element model: (**a**) Straight beam, (**b**) Curve-shaped beam.

At present, most of these existing piezoelectric energy harvesters utilize straight beam as the energy transducing elements due to its advantages in terms of simplicity and ease of fabrication, as shown in Figure 1a.

**Figure 2:**
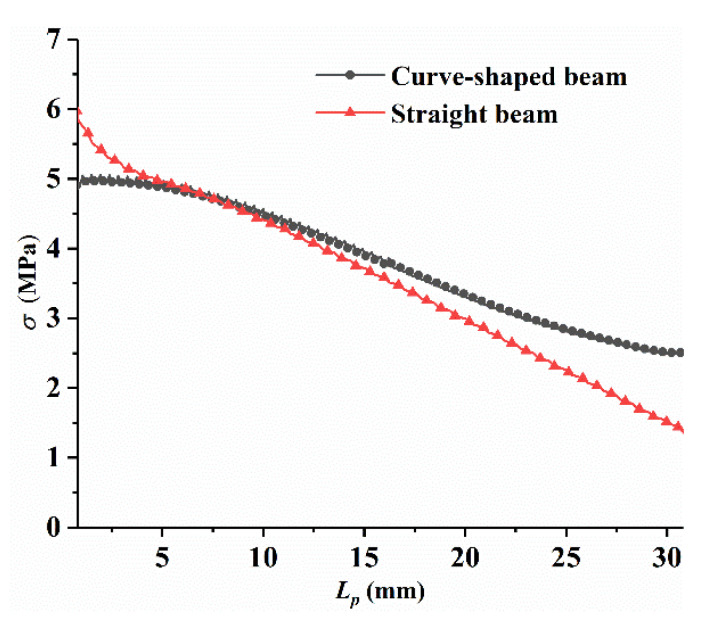
Comparison of stress distribution of PVDF on the curve-shaped beam and the straight beam.

As we know, the conversion efficiency of piezoelectric materials is closely related to the stress distribution of the base layer. The evenly-distributed stress is helpful for harvesting energy and improving conversion efficiency. According to the theory of material mechanics, the conventional straight cantilever experiences a linear stress distribution on the surface when excited. The base layer considered in this work is schematically shown in Figure 1b, which is built of an arc-shaped and a flat configuration, and experiences different stress distribution from the conventional straight cantilever due to the arc-shaped configuration being introduced to improve the stress condition. The finite element analysis was performed in COMSOL software to analyze the influence of curved beam and traditional straight beam structure on the stress distribution of piezoelectric materials (PVDF). In order to make a fair comparison, both beams had the same rectangular sections; the material parameters used are listed in Table 1. Two identical mass were attached at the free end of both beams to reduce resonance frequency, respectively. Note that the curve-shaped beam had an arch with a central angle of 180 degrees, with a radius of *R* = 10 mm. The PVDF was only adhered to the arc-shaped surface of the curve-shaped beam, with a horizontal length of *Lp* = 31.4 mm. Meanwhile, the identical piezoelectric material (PVDF) was attached on the surface of the straight beam. The same load was applied on both beams, respectively. The stress distribution along the length direction of the piezoelectric materials on the curved beam and straight beam is shown in Figure 2, respectively.

It can be seen from Figure 2 that the stress of the piezoelectric material on the straight beam structure decreased linearly from the fixed end. The stress of the piezoelectric material on the curved beam structure was higher than that of the straight beam structure in most areas, and dropped more smoothly than that of the curved beam. The stress distribution was correlated with the bending moment acting on the configuration, the bending moment acting on the straight beam configuration decreased linearly along the fixed end, leading to linearly decreasing stress. However, the bending moment acting on the arc-shaped configuration behaved in a complex manner and decreased nonlinearly along the fixed end according to the theory of material mechanics, thus improving the stress distribution.

### 2.2. Generation Performance Comparisons

The piezoelectric coupling analyses are carried out in COMSOL software to compare the power generation performance of the curved beam and the straight beam structure.

**Figure 3:**
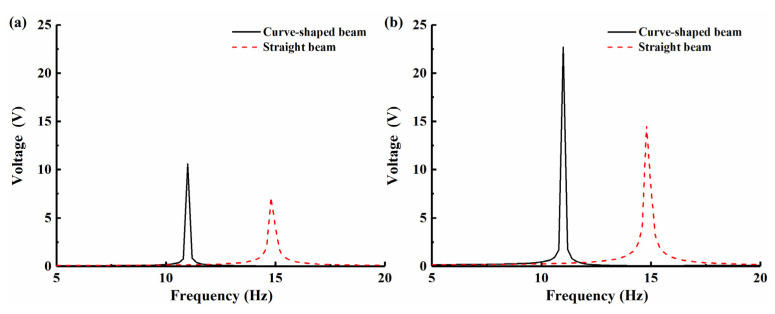
Output voltage obtained by finite-element simulation at different excitation amplitude: (**a**) *A* = 2 m/s^2^; (**b**) *A* = 5 m/s^2^.

Figure 3 shows the voltage comparison diagram of the curved beam and the straight beam structure under two different excitation conditions. At the excitation level of 2 m/s^2^, the resonance voltage of the curved beam was 11 V, and the resonance voltage of the straight beam was 7 V. With an increase in the excitation level to 5 m/s^2^, the resonance voltage of the curved beam was 22 V, and corresponding value of the straight beam was only 15 V in this case. Based on the simulation results, the voltage output of the piezoelectric material on the curved beam structure is always higher than that of the straight beam structure under two different excitation levels. The relatively large and evenly-distributed stress results in less energy dissipation during charge flowing from the large stress region to low, which contributes to enhance the power output and energy conversion efficiency [27,28]. The special stress distribution of the curved beam configuration is beneficial to improving the output performance of the piezoelectric material. Therefore, the piezoelectric material on the surface of the curved beam produces a higher output voltage than that of the straight beam, and the curved beam has a better performance than the straight beam. At the same time, it can be found that curved beams have a lower resonance frequency than the straight beam, which will also benefit energy harvesting in low-frequency environments. Therefore, the introduction of a curved beam structure to a piezoelectric energy harvester is beneficial to increase the output power and improve the output performance of the conventional energy harvester.

## 3. BPEH-V Configuration

**Figure 4:**
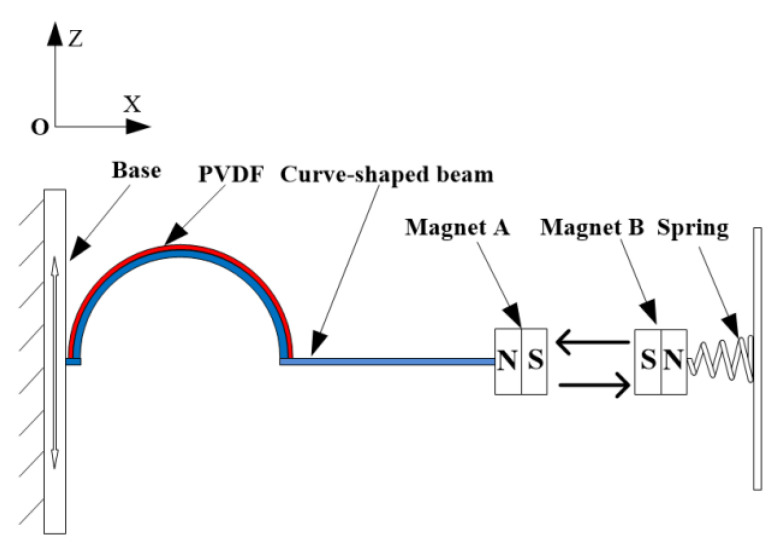
Schematic diagram of the BPEH-V.

The BPEH-V, shown in Figure 4, is comprised of a curve-shaped beam, magnet A, magnet B (i.e., external magnet), piezoelectric material (PVDF), and base. The piezoelectric material is attached to the surface of the arched part of the curve-shaped beam to realize energy conversion, and the flat part remains free. The external magnet B maintains a magnetic repulsive relationship with magnet A, and imposing bistability on the system. The difference between the proposed system and a conventional bistable piezoelectric harvester is because the external magnet B is connected to the base through a spring. If the BPEH-V is excited by ambient vibrations, the piezoelectric cantilever and magnet A are vibrated with the base, so the oscillation of piezoelectric cantilever would result in the deformation of PVDF, thus the conversion of mechanical energy from ambience into electrical energy via the piezoelectric effect can be achieved. When the end magnet of the cantilever beam moves to the intermediate equilibrium position, the spring is compressed and the potential barrier is lowered. Conversely, if the end magnet moves far away from the intermediate equilibrium position, the spring returns to the zero point and the magnetic distance is reduced to maintain the bistable characteristics of the system. Therefore, a bistable piezoelectric energy harvester with variable potential well is formed during the process of the piezoelectric beam vibration.

The BPEH-V not only retains the vibration bistability of the piezoelectric cantilever but could also adjust the potential barrier level, which is beneficial to realizing large-amplitude interwell oscillations under a low excitation level, thus improving the energy harvesting performance.

### 3.1. Theoretical Modeling

#### 3.1.1. Modeling of Nonlinear Restoring Force

**Figure 5:**
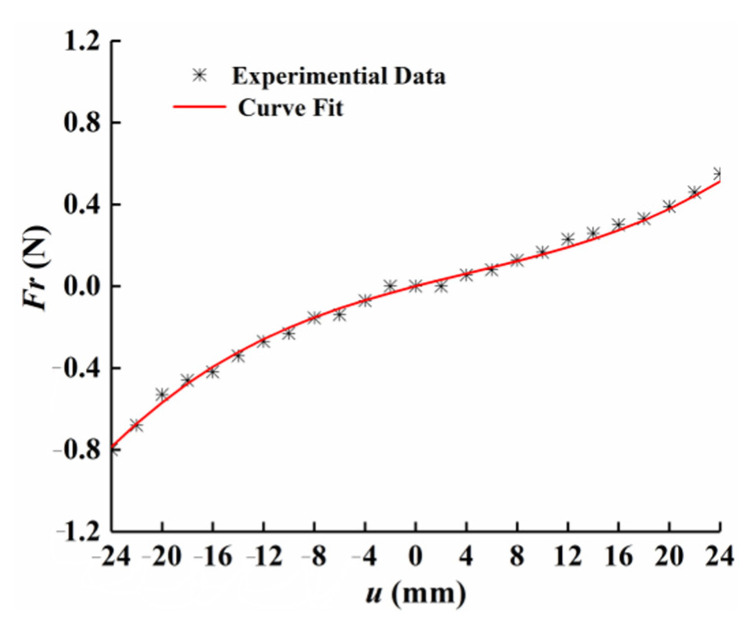
Displacement-restoring force curve of the curve-shaped beam.

Unlike the linear restoring force of the conventional straight beam, the restoring force was nonlinear in the curve-shaped beam due to the existence of the arc-shaped configuration. To model the restoring force, the relationship between deflection and restoring force is extracted by using experimental method. To this end, the curve-shaped beam was fixed on the left end, and the free end of the beam was pushed by the dynamometer to measure the value of the nonlinear restoring force at different displacements. The process was repeated and the measurement results were averaged, then the relationship between the resorting force and transverse displacements were fit to a polynomial, as follows:

(1) $$F_{r}=k_{1}u^{3}\left(L,t\right)+k_{2}u^{2}\left(L,t\right)+k_{3}u\left(L,t\right)$$

where $k_{1}$, $k_{2}$, and $k_{3}$ are constant coefficients on the third, second, and first-order terms, respectively. Figure 5 shows the measurement results and curve fitting results of the nonlinear restoring force of the curve-shaped beam. It can be observed from Figure 5 that the experimental data and the fitting curve had good agreement, and the restoring force of the curve-shaped beam exhibited a curve due to the existence of the curved configuration. Setting *u* = 0 as the static equilibrium position, it was found that the measurement results were asymmetrical, which is due to the fact that the radius of curvature for the curved configuration is continuously varied in the process of the piezoelectric beam vibration, and resulting in asymmetric nonlinear restoring force.

#### 3.1.2. Modeling of Magnetic Force

**Figure 6:**
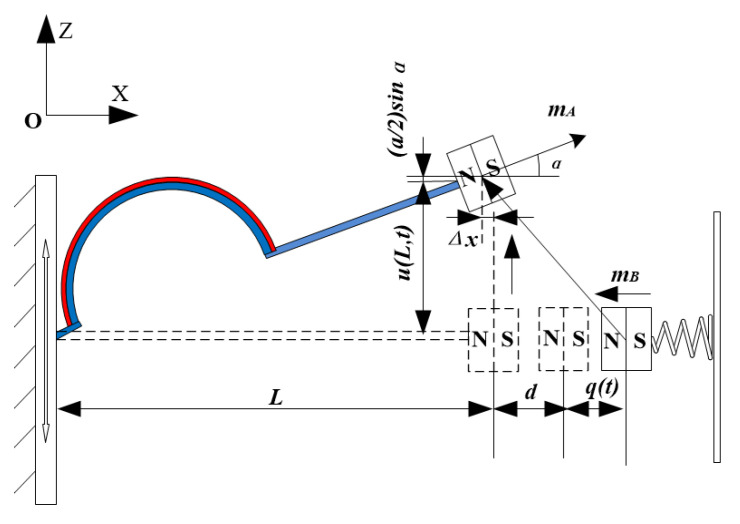
Schematic diagram of the spatial position of the magnets.

The permanent magnets can be modeled as the point dipoles when calculating the magnetic force between the tip magnet and the external magnet. The schematic diagram of the spatial position of the magnets is shown in Figure 6. Considering the additional degree of freedom (DOF) and rotation of the magnet, the distance vector $r_{BA}$ from the center of magnet B to magnet A can be expressed as:

(2) $$r_{BA}=\left[-d-q\left(t\right),\;u\left(L,t\right)\right]$$

where q(t) is the compression displacement of magnet B, and the magnetic field generated by magnet B on magnet A is obtained as [29]:

(3) $$U_{MA}=\frac{\mu_{0}}{4\pi }\left[\frac{m_{B}}{{\left\|r_{BA}\right\|}_{2}^{3}}-\frac{\left(m_{B}\cdot r_{BA}\right)\cdot 3\cdot r_{BA}}{{\left\|r_{BA}\right\|}_{2}^{5}}\right]m_{A}$$

The magnetic moment vectors $m_{A}$ and $m_{B}$ for magnets A and B can be respectively expressed as:

(4) $$m_{A}=\left[M_{A}V_{A}cos\alpha ,\;M_{A}V_{A}sin\alpha \right]$$

(5) $$m_{B}=\left[-M_{B}V_{B},\;0\right]$$

where $M_{i}$ and $V_{i}$ (*i* = A, B) are the magnetization strength and material volume of magnets A and B, respectively. α is the slope of beam at the free end, which is given by:

(6) $$\alpha =arctan\left(\dot{u}\left(L,t\right)\right)$$

Substituting Equation (2) and Equation (4) to Equation (6) into Equation (3), the magnetic field $U_{MA}$ can be expressed in the following equation:

(7) $$U_{MA}=\frac{\mu_{0}M_{A}V_{A}M_{B}V_{B}\left(-u{\left(L,t\right)}^{2}-2{\left(d+q\left(t\right)\right)}^{2}+3\left(d+q\left(t\right))u\left(L,t\right)\dot{u}\left(L,t\right)\right)\right)}{4\pi \sqrt{{\left(\dot{u}\left(L,t\right)\right)}^{2}+1}\;{\left(u{\left(L,t\right)}^{2}+{\left(d+q\left(t\right)\right)}^{2}\right)}^{5/2}}$$

#### 3.1.3. Dynamical Model

To predict the response of BPEH-V, considering the Euler–Bernoulli theory and the linear constitutive equations for piezoelectric materials, the coupled governing equations are derived by using the generalized Hamilton principle.

(8) $$\int_{t1}^{t2}[\delta \left(T_{k}-U_{r}+W_{e}\right)+\delta W_{nc}-\delta U_{m}]=0$$

where $U_{r}$ is the elastic potential energy of the piezoelectric beam and $U_{m}$ is the magnetic potential energy. $W_{e}$ is the electric potential energy of the piezoelectric layer, and $W_{nc}$ is the external work applied to the system. The whole kinetic energy of the proposed system can be expressed as:

(9) $$\;\;\;T_{k}=T_{1}+T_{2}+T_{3}+T_{4}$$

where $T_{1}$, $T_{2}$, $T_{3}$, and $T_{4}$ represent the kinetic energy of the substrate layer, the piezoelectric layer, the tip magnet A, and the movable magnet B.

(10) $$\;T_{k}=\frac{1}{2}\underset{V_{s}}{\int }\rho_{s}{\dot{u}}^{2}\left(x,t\right)dV_{s}+\frac{1}{2}\underset{V_{p}}{\int }\rho_{p}{\dot{u}}^{2}\left(x,t\right)dV_{p}+\frac{1}{2}m_{A}{\dot{u}}^{2}\left(x,t\right){|}_{x=L}\ldots \;+\frac{1}{2}I_{t}{\left[\frac{\partial^{2}u\left(x,t\right)}{\partial t\partial x}{|}_{x=L}\right]}^{2}+\frac{1}{2}m_{B}\dot{q}{\left(t\right)}^{2}$$

where u(x,t) is the transverse displacement of the beam; $V_{p}$ and $V_{s}$ are the piezoelectric and substrate layer volume, respectively; and $I_{t}$ is the rotational inertia of the tip magnet with respect to the beam free end. The electric potential energy of the piezoelectric material can be expressed as follows:

(11) $$\;\;\;\;\;\;\;\;W_{e}=\frac{1}{2}\int_{V_{p}}\varepsilon_{33}^{s}E_{3}^{2}V_{p}+\frac{1}{2}\int_{V_{p}}e_{31}E_{3}S_{1}dV_{p}$$

where $E_{3}$ and $S_{1}$ represent the electrical field and the axial strain, respectively. $\varepsilon_{33}^{s}$ and $e_{31}$ represent the permittivity component at constant strain and the piezoelectric constant. The external work applied to the BPEH-V system can be written as follows:

(12) $$\delta W_{nc}=-\int_{0}^{L}\delta u\left(x,t\right)m\left(x\right)\ddot{z}\left(t\right)dx-\delta u\left(L,t\right)m_{0}\ddot{z}\left(t\right)+Q\delta v$$

In this paper, based on the Rayleigh–Ritz principle, it is assumed that a single-mode approximation of the beam deformation is sufficient, and the vibrational displacement of the beam can be expressed as follows:

(13) $$u\left(x,t\right)=\overset{n}{\underset{i=1}{\sum }}\varphi_{i}\left(x\right)r_{i}\left(t\right)$$

where $\varphi_{i}\left(x\right)$ is the *i*th mode shape of the beam and $r_{i}\left(t\right)$ is the time-dependent generalized coordinates. Under the low frequency excitations, the vibration of the beam is mainly concentrated in the first-order mode, so it is sufficient to consider one mode to obtain the reduced-order model. Meanwhile, for the boundary conditions where one end is clamped and the other one is free, the allowable function can be written as [30,31]:

(14) $$\varphi \left(x\right)=1-cos\left(\frac{\pi x}{2L}\right)$$

Substituting Equations (7) and (10)–(12) into Equation (8), according to Kirchhoff’s law, the governing equations of BPEH-V system are obtained:

(15) $$M\ddot{r}\left(t\right)+C\dot{r}\left(t\right)+k_{1}r^{3}\left(t\right)+k_{2}r^{2}\left(t\right)+k_{3}r\left(t\right)-\frac{\partial U_{MA}}{\partial r\left(t\right)}-\theta v=-H_{s}\ddot{z}\left(t\right)$$

(16) $$\theta \dot{r}\left(t\right)+C_{p}\dot{v}\left(t\right)+\frac{v\left(t\right)}{R}=0$$

(17) $$m\ddot{q}\left(t\right)+kq\left(t\right)-F_{q}=0$$

where *M* and C refer to the mass coefficient and the damping coefficient, respectively. θ is the electromechanical coupling coefficient; $C_{p}$ is the capacitance of the piezoelectric patch; R is the load resistance; and $F_{q}$ is the horizontal magnetic force component, as follows:

(18) $$M=\int_{\Omega b}\rho_{b}\varphi {\left(x\right)}^{2}d_{\Omega b}+\int_{\Omega p}\rho_{p}\varphi {\left(x\right)}^{2}d_{\Omega p}+m_{0}\varphi {\left(L\right)}^{2}+I_{t}\varphi^{\prime 2}\left(L\right)$$

(19) $$\;H_{s}=\rho_{b}A_{b}\int_{0}^{L}\varphi \left(x\right)dx+\rho_{p}A_{p}\int_{0}^{L_{p}}\varphi \left(x\right)dx+m_{0\;}$$

(20) $$C=\int_{0}^{L}c\varphi {\left(x\right)}^{2}dx$$

(21) $$\theta =\frac{1}{h_{p}}\int_{\Omega p}e_{31}z\varphi^{\prime \prime }\left(x\right)d_{\Omega p}$$

(22) $$C_{p}=\frac{\varepsilon_{33}^{S}b_{p}L_{p}}{h_{p}}$$

(23) $$F_{q}=\frac{\partial U_{MA}}{\partial q\left(t\right)}$$

## 4. Numerical Simulation

### 4.1. Study on the Potential Energy of BPEH-V

**Figure 7:**
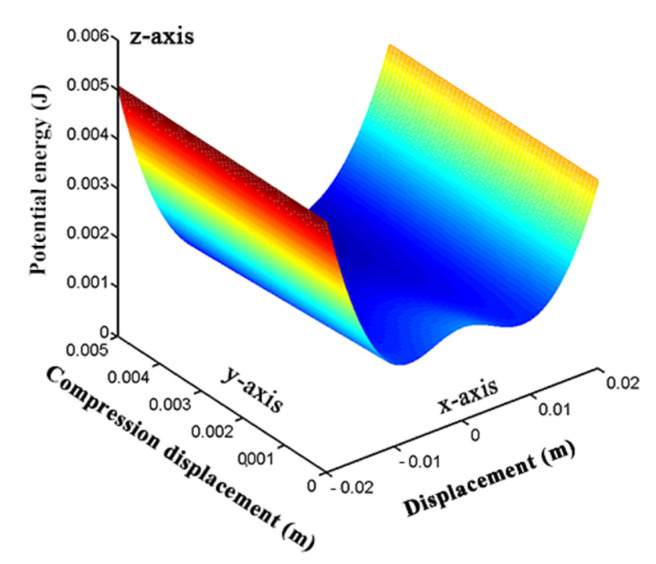
Potential curve of the system at different compression displacement of the spring.

Magnetic potential energy is an important factor that affects the nonlinearity of the system. Different magnetic distances will produce different nonlinear magnetic forces, so the system presents different characteristics. Regarding the BPEH-V system, the magnetic potential energy is continuously varied with vibration due to the external magnet being connected elastically. Figure 7 shows the potential energy curve of the system under the condition of magnetic distance (*d* = 17 mm). In this case, two obvious potential wells are formed, that is, the system becomes bistable. We should notice that magnetic distance *d* is constantly varied during the vibration of the piezoelectric cantilever beam, so the potential energy of the system is different from a traditional bistable piezoelectric energy harvester with a fixed external magnet. The magnetic potential energy is not only affected by the magnetic distance *d*, but also by the compression displacement *q*(*t*) of the spring. As shown in Figure 7, the *x*-axis denotes displacement of the curved-shape beam’s tip, the *y*-axis denotes the compression displacement of the spring, and the *z*-axis denotes the potential energy of the system. The height of the barrier between the two wells is pulled down as the compression displacement of the spring gradually increases due to the repulsive force between the tip magnet and the external magnet. In this condition, the system can cross the potential barrier to realize interwell oscillations more easily. The influence of *q*(*t*) in BPEH-V, which is caused by spring compression, equals that of time-varying *d* in the traditional bistable system. When the tip magnet tends to approach its original point (at u(L,t)=0 in Figure 6), it drives the external magnet away from the equilibrium position due to magnetic repulsion, thus decreasing the potential barrier. Conversely, when the tip magnet moves far away from the original point, the potential barrier gradually becomes high and reaches its maximum. Thus, the design of the BPEH-V provides an adaptive potential using the spring in comparison to the traditional bistable system.

Meanwhile, it can also be seen from Figure 7 that the potential energy curve of the proposed system is inconsistent with the straight beam bistable piezoelectric energy harvester. The potential well is shallower on the left side and deeper on the right side, showing an asymmetrical trend. This is mainly due to the asymmetric restoring force of the curve-shaped beam.

### 4.2. The Dynamics Analysis of BPEH-V

According to the potential energy diagram shown in Figure 7, the system becomes bistable and the height of the potential barrier is relatively shallow when the magnet distance is *d* = 17 mm. In this section, the numerical simulations are performed for the separation distance *d* = 17 mm to investigate the influence of the variable potential well on the dynamic characteristics of BPEH-V (the ode45 command of MATLAB was used here).

**Figure 8:**
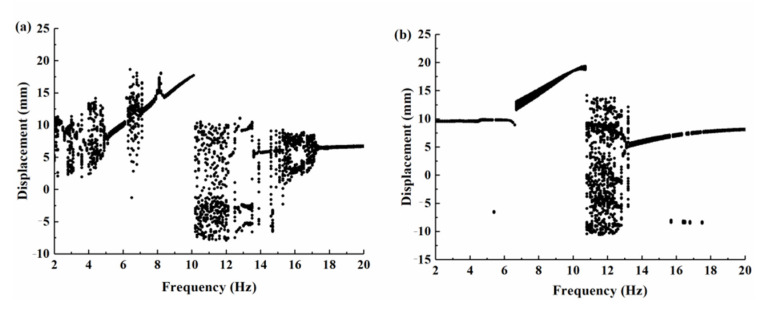
Bifurcation diagram of the tip displacement versus the excitation frequency for excitation amplitude *A* = 10 m/s^2^: (**a**) BPEH-V; (**b**) CBH-C.

**Figure 9:**
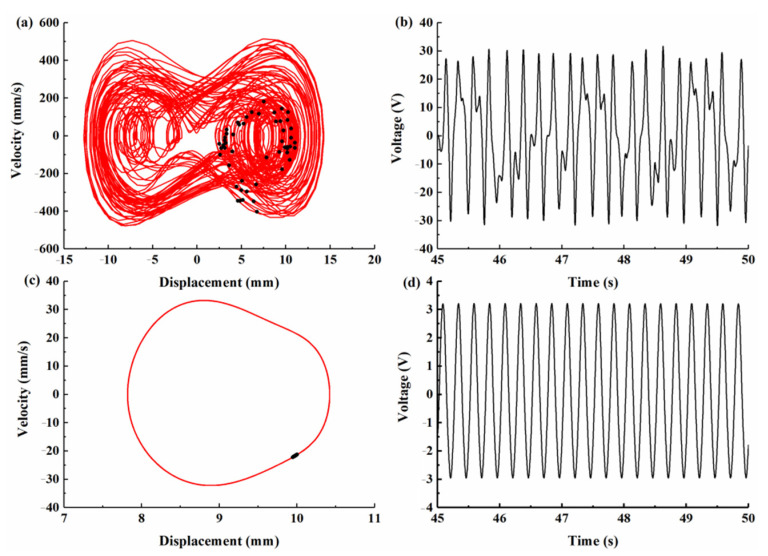
Phase plane portrait, Poincaré map, and output voltage histories for excitation frequency *f* = 4 Hz. (**a**) Phase plane portrait and Poincaré map. (**b**) Voltage histories for BPEH-V, respectively. (**c**) Phase plane portrait and Poincaré map. (**d**) Voltage histories for CBH-C, respectively.

The bifurcation diagram of the tip displacement versus the excitation frequency of the BPEH-V and the CBH-C for excitation amplitude *A* = 10 m/s^2^ is shown in Figure 8. Compared to Figure 8a,b, it can be found that BPEH-V exhibited more complex dynamic behaviors than the CBH-C. At 4 Hz excitation, BPEH-V enters into the chaotic oscillation, which can be concluded from the phase plane portrait (the phase plane portrait is drawn by red curves) and Poincaré map (the Poincaré map is drawn by black dots) depicted in Figure 9a.

However, the CBH-C system only made a small-amplitude intrawell motion at this time, as shown in Figure 9c. Meanwhile, compared with Figure 9b,d, we found that the BPEH-V generated a much higher output voltage than the CBH-C in the low excitation frequency.

**Figure 10:**
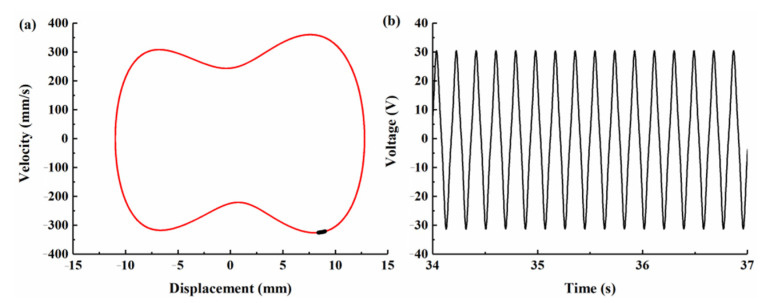
Simulation results for BPEH-V under excitation frequency *f* = 5 Hz. (**a**) Phase plane portrait and Poincaré map. (**b**) Output voltage histories.

With the increase in excitation frequency to 5 Hz (Figure 10 shows the simulation results for BPEH-V), the Poincaré map is concentrated in a single point and the phase plane portrait consists of a closed obit, as shown in Figure 10a, which demonstrates that the BPEH-V entered into large-amplitude periodic oscillations. However, the CBH-C system still made a small-amplitude intrawell motion at this time, as can be found from the bifurcation diagram of the tip displacement versus the excitation frequency depicted in Figure 8b.

**Figure 11:**
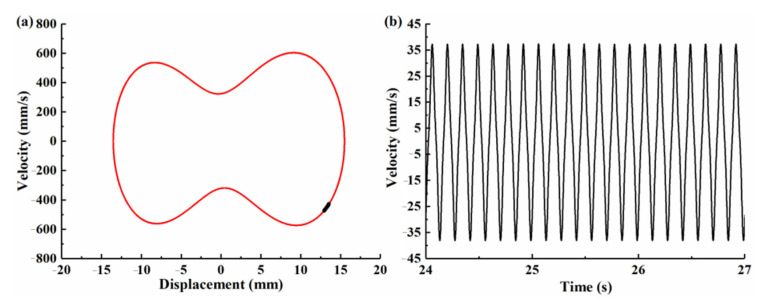
Simulation results for CBH-C under excitation frequency *f* = 7.1 Hz. (**a**) Phase plane portrait and Poincaré map. (**b**) Output voltage histories.

With the increase in excitation frequency to 7.1 Hz, Figure 11 shows the simulation results for CBH-C, where the Poincaré map is concentrated in a single point and the phase plane portrait consists of a closed obit, which demonstrates that the CBH-C enters into large-amplitude periodic oscillations. Meanwhile, it was observed from Figure 8a that the BPEH-V underwent transient chaotic oscillation at 6.3 Hz excitation, and then returned to large-amplitude interwell oscillations at 7 Hz excitation.

With the still further increase in excitation frequency, the BPEH-V exits large-amplitude interwell oscillations when the excitation frequency exceeds 10.1 Hz. Meanwhile, the CBH-C exits large-amplitude interwell oscillations at a frequency *f* = 10.3 Hz.

From the above simulations and analyses, we found that the frequency ranges of large-amplitude periodic response of BPEH-V were 5 < *f* < 6.3 Hz and 7 < *f* < 10.1 Hz, and the effective bandwidth was 4.4 Hz. The corresponding frequency range of CBH-C was 7.1 < *f* < 10.3 Hz, and the effective bandwidth was only 3.2 Hz. Accordingly, the effective bandwidth of BPEH-V was 1.37 times that of CBH-C under the same circumstances due to the spring being efficiently introduced to broaden bandwidth, and the BPEH-V was superior to the CBH-C from the aspect of effective bandwidth. The conventional bistable system only made a small-amplitude intrawell motion at low excitation frequency due to the lack of sufficient energy to overcome the potential barrier. However, thanks to the compression adjustment of the spring, it can pull down the potential barrier and form an adaptive potential barrier. The BPEH-V with suitable stiffness can realize large-amplitude interwell motions at the lower excitation frequency, thus improving the harvesting performance.

### 4.3. The Influence of the Spring Stiffness K on Harvesting Performance

**Figure 12:**
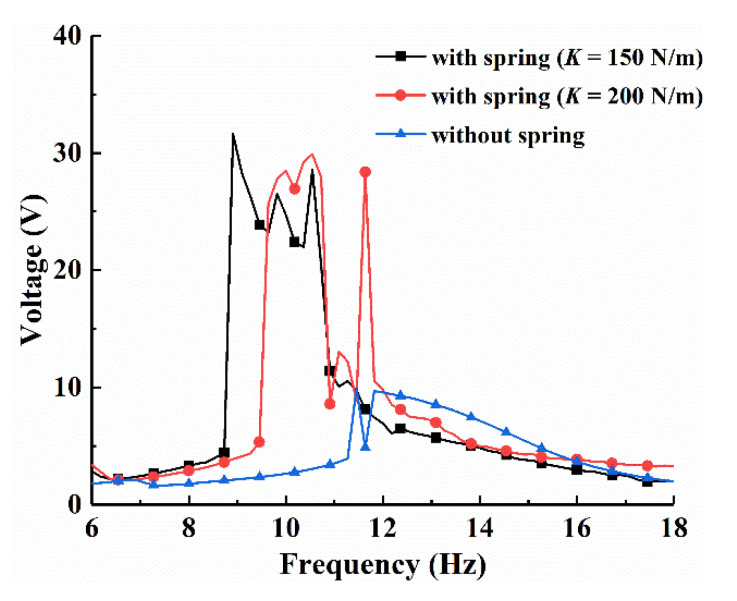
Frequency-swept voltage response of the system with different spring stiffness under excitation amplitude *A* = 5 m/s^2^.

Spring stiffness has a great impact on the system characteristics. In order to investigate the influence of the spring stiffness *K* on energy harvesting performance, the numerical frequency-swept experiments of the BPEH-V system with three distinct spring stiffness were conducted under the excitation amplitude of 5 m/s^2^, as shown in Figure 12. The BPEH-V system with suitable stiffness of *K* = 200 N/m can realize large-amplitude interwell oscillations and have a higher output at a frequency range of *f* = 9.4–11Hz. We decreased the spring stiffness to *K* = 150 N/m. The spring was more easily compressed due to the small spring stiffness, so the system could realize large-amplitude interwell oscillations at lower excitation frequency; the theoretical frequency range of the large-amplitude periodic response was *f* = 8.6–11 Hz; and the effective bandwidth was 2.4 Hz, which was broader than the case of *K* = 200 N/m. Meanwhile, we notice that the system without spring can only realized intrawell oscillations and generated a lower output voltage at the same condition, which was because that the system could not obtain sufficient energy at low excitation level to overcome the potential barrier, thus resulting in poor output performance. Therefore, the BPEH-V with suitable spring stiffness contributed to realize large-amplitude interwell oscillations over a wide range of excitation, especially in low excitation level compared to CBH-C.

It needs to be mentioned that we should ensure the bistable characteristic of the system when choosing a small stiffness spring as the connection element. Otherwise, the system will be close to a linear one and vibrates around the middle equilibrium point, leading to poor output performance.

## 5. Conclusions

This paper proposed a magnetically coupled bistable piezoelectric energy harvester based on an elastically connected external magnet. First, finite-element simulations were performed for the curve-shaped composite and the straight beam to compare the influence of different configurations on the stress distribution and power generation performance. Moreover, the dynamics model of the system was established by using the generalized Hamilton variational principle, and the fourth-order Runge–Kutta algorithm was used to numerically solve the dynamic equations. The dynamic characteristics of the piezoelectric energy harvester were analyzed and compared with the traditional curve-shaped beam bistable harvester. Finally, the influence of the spring stiffness on energy harvesting performance of the system was discussed. The main conclusions are as follows:

1. The curve-shaped configuration beams had a larger and more uniform strain distribution than the straight beam due to the special arched structure. Under the same excitation conditions, compared with the traditional straight beam, the curve-shaped configuration beam had a higher output voltage. Therefore, the curve-shaped beam was introduced into the nonlinear piezoelectric energy harvester, which can help to improve the harvesting efficiency of the energy harvesting device.
2. A spring was connected with an external magnet to form an elastically supported bistable system. The potential energy of the system was affected by the magnetic distance and spring stiffness. The elastic connection of the external magnet could adjust the height of the system’s barrier to realize an adaptive potential barrier. Compared with the rigidly connected bistable system, the elastically connected system can makes large-amplitude oscillations easier, which is beneficial to improve the performance of the energy harvester, especially suitable for energy harvesting in a low frequency environment.
3. The spring stiffness has an important effect on the performance of the proposed system. A spring with a small spring stiffness is beneficial for the system to achieve a large-amplitude oscillation over a wider frequency band. However, in practical applications, the spring stiffness affects the position of the equilibrium points of the system, the minimum spring stiffness must be able to maintain the bistable characteristics of the system, which is a problem that must be considered in the design. Otherwise, the elastically connected bistable system will lose its bistable characteristics and degenerate into a nonlinear monostable system, thus resulting in poor energy harvesting performance.

In addition, experimental investigations will be presented in the future.

## Figures and Tables

**Table 1:** Material parameters for simulation.

Parameter	Symbol	Value	**Unit**
Substrate layer (beryllium bronze)			
Density	*ρ_s_*	8300	kg/m^3^
Elastic modulus	*E_s_*	128	GPa
Arc-shaped radius	*R_s_*	10 × 10^−3^	m
Horizontal length	*L_s_*	40 × 10^−3^	m
Height	*h_s_*	2 × 10^−4^	m
Width	*b_s_*	8 × 10^−3^	m
Piezoelectric layer (PVDF)			
Density	*ρ_p_*	1780	kg/m^3^
Elastic modulus	*E_p_*	3	GPa
Length	*L_p_*	31.4 × 10^−3^	m
Height	*h_p_*	1.1 × 10^−4^	m
Width	*b_p_*	8 × 10^−3^	m
Piezoelectric stress constant	${\bar{e}}_{31}$	11.5	C/m^2^

## Data Availability

Not applicable.
